# Long Esophageal Stricture in a Brittle Diabetic

**DOI:** 10.5005/jp-journals-10018-1248

**Published:** 2017-09-29

**Authors:** Stella C Pak, Umar Darr, Yaseen Alastal, Youngsook Yoon

**Affiliations:** 1 Department of Medicine, University of Toledo Medical Center, Toledo, Ohio, USA

**Keywords:** Diabetes mellitus, Esophageal dilation, Esophageal stricture, Gastroesophageal reflux disease, Subtotal esophagectomy.

## Abstract

**Aim:**

We report a case of atypical esophageal stricture in a young diabetic woman.

**Background:**

Diabetes mellitus and gastroesophageal reflux disease (GERD) are two common disorders in modern society.

**Case report:**

A young diabetic woman developed a 6-cm-long esophageal stricture. This stricture was refractory to multiple esophageal dilation procedures. She underwent subtotal esophagectomy and had excellent treatment outcome.

**Conclusion:**

Gastroesophageal reflux disease can cause severe long esophageal stricture in a brittle diabetic.

**Clinical significance:**

Improving the awareness of their association between diabetes and GERD would greatly benefit the day-to-day practice of medicine.

**How to cite this article:** Pak SC, Darr U, Alastal Y, Yoon Y. Long Esophageal Stricture in a Brittle Diabetic. Euroasian J Hepato-Gastroenterol 2017;7(2):191-192.

## BACKGROUND

The prevalence of diabetes mellitus is estimated to be about 1 in every 11 people in the United States.^1^ Hyperglycemia in diabetic patients disturbs the delicate neurological cascades in the gastrointestinal (GI) system. Microvascular damage in the myenteric plexus in diabetes further exacerbates the neurological balance.^2^ The neurological balance often results in esophageal dysmotility, gastroparesis, diarrhea, constipation, and fecal incontinence. Gastrointestinal complications worsen postprandial glycemic fluctuation. Therefore, diabetes and its GI complications are chained in a loop, perpetuating each other.

Gastroesophageal reflux disease is also a very common disorder, with prevalence of approximately 1 in every 4 people in the United States.^3^ Intestinal motility dysfunction in diabetes predisposes patients to the development of GERD. As a result, diabetics are 1.25 times more likely to have GERD than the general population. Therefore, improving the awareness in the association between diabetes and GERD is critical in modern day practice.

A known complication of GERD is short esophageal strictures, under 2 cm, that can be managed with acid sup-pression therapy or endoscopic dilation.^4,5^ Herein, we report a 27-year-old diabetic who developed a 6 cm peptic stricture from GERD. She underwent partial esophagectomy.

## CASE REPORT

A 27-year-old brittle diabetic female presented with 3 years duration of worsening dysphagia accompanied by nonbloody vomiting and severe malnutrition. These symptoms persisted despite multiple dilation procedures with mechanical balloon and push dilator (Savary-Gilliard dilator). Her medical history was significant for type 1 diabetes mellitus complicated by gastroparesis and multiple episodes of diabetic ketoacidosis. She also suffered from GERD for the past 5 years.

At the time of admission, her height, weight, and body mass index (BMI) were 155.4 cm, 32.2 kg, and 13.3 respectively. Her hemoglobin was 7.7 g/dL and prealbumin was 8.7 mg/dL. In the view of severe malnutrition, a jejunostomy tube (J-tube) was placed for enteral feeding. She tolerated J-tube feeding well.

**Fig. 1:**
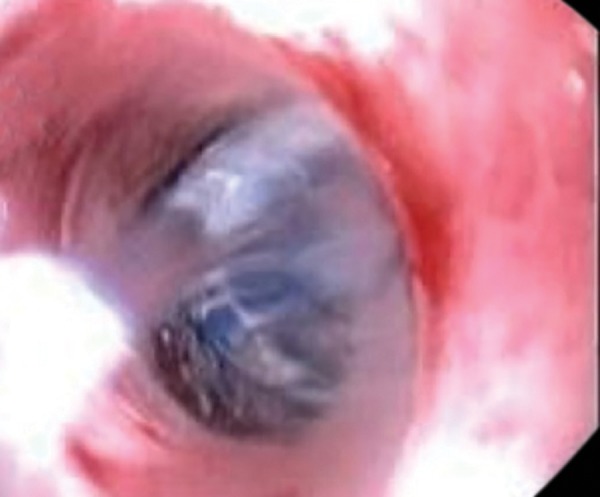
A stricture at esophagus

**Fig. 2:**
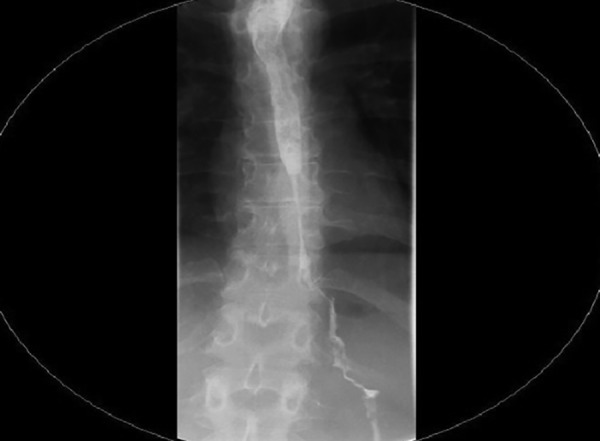
Barium meal assessment of stricture

Endoscopic examination revealed severe erosive esopha-gitis with overlying exudate, mainly over the lower third of the esophagus. A severe stricture, measuring 60 mm along the longitudinal axis, located 29 to 35 cm from the gastroesophageal junction, was noted (Fig. 1). Barium swallow study also visualized the long peptic stricture (Fig. 2).

Since dilation procedures failed to resolve the stricture, McKeown esophagectomy was performed through combined abdominothoracic approach. During the operation, a tremendous amount of scarring was identified in the periesophageal plane. The thoracic segment of esophagus, and fundus, cardia, and body segments of stomach were removed. Visual examination of the esophagus revealed deep mucosal erosion extending down to the muscularis propria with associated granulation tissue. The mucosa within the stricture site had an ulcerating hemorrhagic appearance. Pyloroplasty was also performed given her history of chronic gastroparesis and diabetes, increasing the likelihood of severe postoperative gastroparesis.

She had uneventful postoperative recovery and was discharged on 20th day of hospitalization. After discharge, she gradually transitioned from tube feeding to oral feeding over 1 month. At present, 1 year and 2 months after surgery, she is tolerating oral intake. Her current BMI, hemoglobin, and prealbumin are 14.5, 10.9 g/dL, and 9.6 mg/dL respectively.

## DISCUSSION

First line of management for esophageal stricture is acid suppression therapy using proton pump inhibitors or histamine antagonists.^4^ Alternative conservative management is dilation procedure using push or balloon dilators. Push dilators can be either weighted or wire guided. The mostly widely used push dilator is the polyvinyl tube (Savary-Gilliard dilator). Balloon dilators can be passed through the scope or wire guided.^6^ The atypical peptic stricture in our patient was refractory to both acid suppression therapy and dilation procedures.

Least invasive surgical approach is the resection of esophageal segment. Subtotal esophagectomy is a more invasive procedure reserved for treatment for severe peptic strictures or strictures with malignancy potential.^4^

In our patient, subtotal esophagectomy was performed due to the severity of refractory peptic strictures.

The vast majority of esophageal strictures associated with GERD tend to be shorter than 2 cm and not extend beyond 4 cm from the gastroesophageal junction.^5^ The size, location, and the extent of clinical manifestation of this esophageal stricture in our patient were unique. The therapeutic challenge associated with this atypical esophageal stricture was also discussed in the present case report.

## CONCLUSION

In summary, we presented a case exemplary for successful management of atypical and refractory stricture in the esophagus of a diabetic patient. As diabetes and GERD are very common diseases, it is critical for clinicians to be aware of their association. Left untreated, esophageal strictures can cause esophageal perforation and malnutrition. Thus, timely and proper treatment should be provided to patients presenting with esophageal strictures.

## CLINICAL SIGNIFICANCE

Gastroesophageal reflux disease can cause severe long esophageal stricture in a brittle diabetic.

## References

[1] Egan E (2017). Diabetes-Related Microvascular Complications: What Every Nurse Practitioner Needs to Know. J Nurse Pract.

[2] Punjabi P, Hira A, Prasad S, Wang X, Chokhavatia S (2015). Review of gastroesophageal reflux disease (GERD) in the diabetic patient. J Diabetes.

[3] Herregods TV, Bredenoord AJ, Smout AJ (2015). Pathophysiology of gastroesophageal reflux disease: new understanding in a new era. Neurogastroenterol Motil.

[4] Matsumoto A, Omura N, Ishibashi Y, Suzuki Y, Nakada K, Kashiwagi H, Yanaga K (2007). Esophageal stricture caused by reflux esophagitis requiring subtotal esophagectomy: a case report. Esophagus.

[5] Yamasaki Y, Ozawa S, Oguma J, Kazuno A, Ninomiya Y (2016). Long peptic strictures of the esophagus due to reflux esophagitis: a case report. Surg Case Rep.

[6] Riley SA, Attwood SEA (2004). Guidelines on the use of oesophageal dilatation in clinical practice. Gut.

